# Cardiovascular Pharmacological Support Among Preterm Infants in Chinese Referral Center Neonatal Intensive Care Units

**DOI:** 10.3389/fped.2021.638540

**Published:** 2021-04-22

**Authors:** Ningxin Luo, Siyuan Jiang, Patrick J. McNamara, Xiaoying Li, Yan Guo, Yang Wang, Junyan Han, Yingping Deng, Yi Yang, Shoo K. Lee, Yun Cao

**Affiliations:** ^1^Children's Hospital of Fudan University, Shanghai, China; ^2^Department of Pediatrics and Internal Medicine, University of Iowa, Iowa City, IA, United States; ^3^Qilu Children's Hospital of Shandong University, Jinan, China; ^4^Children's Hospital of Nanjing Medical University, Nanjing, China; ^5^The First Affiliated Hospital of Anhui Medical University, Hefei, China; ^6^National Health Commision (NHC) Key Laboratory of Neonatal Diseases (Fudan University), Children's Hospital of Fudan University, Shanghai, China; ^7^Maternal-Infant Care Research Centre and Department of Pediatrics, Mount Sinai Hospital, Toronto, ON, Canada; ^8^Department of Pediatrics, University of Toronto, Toronto, ON, Canada; ^9^Department of Obstetrics and Gynecology, Dalla Lana School of Public Health, University of Toronto, Toronto, ON, Canada

**Keywords:** preterm infants, neonate, cardiovascular pharmacological support, neonatal intensive care units, China, outcome assessment

## Abstract

**Objective:** To describe cardiovascular pharmacological support in infants born at <34 weeks' gestation within the first postnatal week in Chinese neonatal intensive care units (NICUs).

**Design:** A secondary analysis of data from a multicenter randomized controlled study (REIN-EPIQ). A questionnaire regarding cardiovascular support practices was also completed by all participating NICUs.

**Setting:** Twenty-five tertiary hospitals from 19 provinces in China.

**Patients:** All infants born at <34 weeks' gestation and admitted to participating NICUs within the first postnatal week from May 2015 to April 2018 were included. Infants who were discharged against medical advice were excluded.

**Measures and Main Results:** Among the 26,212 preterm infants <34 weeks, 16.1% received cardiovascular pharmacological support. The use rates increased with decreasing gestational age and birth weight, with 32.5% among infants <28 weeks and 35.9% among infants <1,000 g. Cardiovascular pharmacological support was independently associated with higher risks of death (aOR 2.8; 95% CI 2.4–3.3), severe intraventricular hemorrhage (IVH) (aOR 2.1; 95% CI 1.8–2.5) and bronchopulmonary dysplasia (BPD) (aOR 2.2; 95% CI 2.0–2.5). Overall 63.1% courses of cardiovascular pharmacological support were >3 days. Prolonged cardiovascular pharmacological support (>3 days) was independently associated with lower rates of survival without morbidity in very-low-birth-weight infants, compared with infants with shorter durations. Dopamine was the most commonly used cardiovascular agent. The cardiovascular pharmacological support rates varied from 1.9 to 65.8% among the participating NICUs.

**Conclusions:** The rate of cardiovascular pharmacological support within the first postnatal week was high with prolonged durations in Chinese NICUs. Marked variation in cardiovascular support existed among participating NICUs. Cardiovascular pharmacological support during the early postnatal period, especially prolonged, may be associated with adverse neonatal outcomes.

**Clinical Trial Registration:** The original trial was registered as “Reduction of Infection in Neonatal Intensive Care Units using the Evidence-based Practice for Improving Quality” (ID: NCT02600195) on clinicaltrials.gov. https://clinicaltrials.gov/ct2/show/NCT02600195?term=NCT02600195&draw=2&rank=1.

## Background

Cardiovascular pharmacological support is among the most commonly used therapies in NICUs, especially in preterm infants (1). The first few days after birth represent a unique period of life as the dynamics of the circulatory system change for adaptation to an extra-uterine environment (2). During this vulnerable period, preterm infants are at high risk of systemic hypotension. The sudden increase in systemic vascular resistance leading to transient myocardial dysfunction, which prompts the need for cardiovascular pharmacological support, is thought to be a major contributor to low cardiac output and suboptimal oxygen delivery to the brain (3). There is no consensus, however, on the definition of an acceptable blood pressure; therefore, wide variations in intervention thresholds, medication selection, duration, and dosage of cardiovascular pharmacological support exist (4, 5). In addition, it is unclear whether cardiovascular pharmacological support during the early stage of life could improve the outcomes of preterm infants (6–9). Data on cardiovascular pharmacological support in Chinese NICUs and outcomes among infants who received drugs have not been reported. The primary aim of this study was to describe the use of cardiovascular pharmacological support during the first postnatal week among the largest contemporary cohort of infants born at <34 weeks gestation in Chinese NICUs.

## Methods

### Study Design

The study was a secondary analysis of data from a multicenter cluster randomized controlled study named “Reduction of Infection in Neonatal Intensive Care Units using the Evidence-based Practice for Improving Quality (REIN-EPIQ),” which was registered at clinicaltrials.gov (NCT0260015) (10, 11). Twenty-five tertiary hospitals from 19 provinces in China participated in this study. These tertiary NICUs represent the highest level of neonatal care in their regions. The study was approved by the Ethics Committee of the Children's Hospital of Fudan University [approval number (2015) 28] for all sites.

### Study Population

All infants born at <34 weeks gestation between May 1st, 2015, and April 30th, 2018, and admitted to 25 participating NICUs within the first postnatal week were enrolled. Infants who were discharged against medical advice and did not receive complete care within the first postnatal week were excluded.

### Data Collection

Detailed clinical data, including data regarding duration of cardiovascular pharmacological support, were collected prospectively by trained data abstractors using a standardized database. All data collection followed standard operations and definitions.

### Variable Definitions

Cardiovascular pharmacological support was defined as continuous intravenous infusion of at least one of the following medications: dopamine, dobutamine, epinephrine, norepinephrine or milrinone. Detailed information, such as the dose of the medication, was not collected. The transport risk index of physiologic stability (TRIPS) score (12), a critical illness score developed by the Canadian Neonatal Network, was used to represent the illness severity on admission. Early onset sepsis (EOS) was defined as sepsis occurring in 3 days of age. Extremely pre-mature infants (EPI) and very pre-mature infants were defined as infants born with gestational age <28^+0^ weeks and 28^+0^-31^+6^ weeks, respectively. Very-low-birth-weight (VLBW) and Extremely-low-birth-weight (ELBW) infants were defined as infants born with a birth weight <1,500 and <1,000 g, respectively.

### Outcomes

The primary outcome was a composite of one of six major morbidities including periventricular leukomalacia (PVL), intraventricular hemorrhage (IVH), retinopathy of pre-maturity (ROP), bronchopulmonary dysplasia (BPD), late onset sepsis (LOS) or necrotizing enterocolitis (NEC). “Survival without morbidity” was defined as surviving infants who were not diagnosed with any of these major morbidities at discharge. PVL was defined as the presence of periventricular cysts on cranial ultrasound or magnetic resonance imaging scans before discharge. IVH was detected by ultrasound findings and was defined according to the criteria of Papile et al. (13). Severe IVH was defined as grade III or IV IVH. ROP was defined according to the International Classification of ROP (14). Severe ROP was defined as ROP ≥ stage 3. BPD was defined as mechanical ventilation or oxygen dependency at 36 weeks post-menstrual age or discharge (15). LOS was defined as blood culture-proven sepsis occurring after 3 days of life (16, 17). NEC was defined as stage II or above NEC according to Bell's criteria (18). Survival without morbidity was defined as infants who survived at discharge without being diagnosed with any of the six major morbidities.

### Survey of Intervention Thresholds and Cardiovascular Pharmacological Support

To evaluate clinical practices of cardiovascular pharmacological support for preterm infants in different NICUs, a questionnaire was developed and filled out as a specific addition by the NICU director or a senior neonatologist according to the practice guidelines (if existed) or general practices in the individual NICU. The contents of the questionnaire included (i) the diagnostic criteria of hypotension; (ii) the most commonly used cardiovascular pharmacological support; (iii) indications for cardiovascular pharmacological support; (iv) indications for discontinuing cardiovascular pharmacological support; (v) methods of blood pressure measurement and circulation status evaluation. The questionnaire can be found as Supplementary Material.

### Statistical Analysis

Infant characteristics and outcomes were compared between the cardiovascular pharmacological support and control groups by Student's *t*-tests for continuous variables and Chi-square-tests for categorical variables. A multivariate logistic regression model was used to compare outcomes between the two groups adjusting for relevant confounders, including gestational age, male sex, being small for gestational age, 1-min Apgar score ≤3 min, cesarean section, antenatal steroids, prenatal care, maternal hypertension, maternal diabetes, primigravida, inborn status and TRIPS score. Variations in cardiovascular pharmacological support among the participating NICUs were also examined by multivariate logistic regression adjusting for the same relevant confounders. An important consideration was the fact that the duration of cardiovascular pharmacological support could be abbreviated by death. Therefore, we performed additional subgroup analyses to compare morbidity rates in survivors who received cardiovascular pharmacological support for ≤3 days and those who received therapy for >3 days. Separate analyses were performed for extremely-low-birth-weight (ELBW) infants (birth weight <1,000 g), very-low-birth-weight (VLBW) infants (birthweight 1,000–1,500 g) and infants with birth weights ≥1,500 g. Stata version 15.0 (StataCorp LP, USA) was used for statistical analysis. Statistical significance was set as a *p*-value of <0.05.

## Results

### Infant Characteristics

A total of 27,532 preterm infants born at a gestational age <34 weeks were admitted to participating NICUs during the study period. After excluding 1,320 infants who were discharged against medical advice within the first week of life, the remaining 26,212 infants constituted the study population. The median gestational age and weight at birth were 31.3 (IQR: 30.1–33.0) weeks and 1,629 (IQR: 1,340–1,900) grams, respectively. Extremely pre-mature infants (EPI, <28 weeks) and ELBW infants accounted for 6.2% (1,622) and 5.4% (1,432) of the sample, respectively.

### Cardiovascular Pharmacological Support Rate

**Figure 1:**
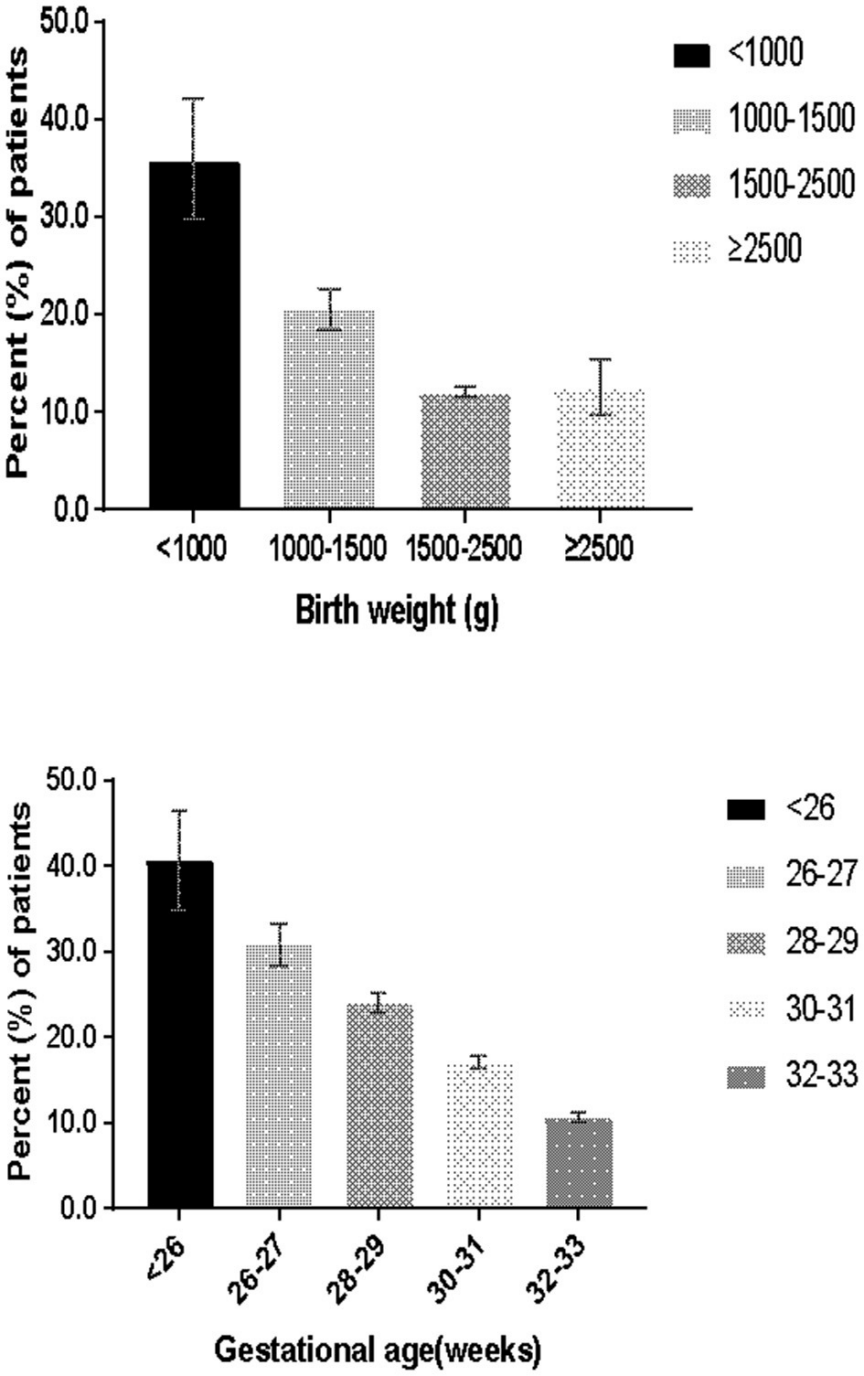
Percentage of patients receiving cardiovascular pharmacological support.

Overall, 16.1% (4,226/26,212) of infants received cardiovascular pharmacological support during the first week. The rates of cardiovascular pharmacological support in EPI and ELBW infants were 32.5% (527/1,622) and 35.9% (514/1,432), respectively. The proportion of preterm infants who received cardiovascular pharmacological support decreased with increasing gestational age and birth weight (Figure 1). Overall, 89.6% (3,788/4,226) of cardiovascular pharmacological support were initiated within 3 days, and 58.4% (2,470/4,226) were initiated on the first day.

Infants who received cardiovascular pharmacological support had lower gestational ages, lower birth weights, lower 1 and 5-min Apgar scores and higher TRIPS scores than infants who were not treated with these drugs. Infants receiving cardiovascular pharmacological support were also more likely to be male and to be small for their gestational age (Table 1).

**Table 1:** Characteristics of the study population.

**Characteristics**	**Cardiovascular pharmacological support group,** **(*N* = 4,226)**	**No cardiovascular pharmacological support group,** **(*N* = 21,986)**	***p*-value**
Gestational age (weeks), median (IQR)	30.5 (29.0–32.3)	31.5 (30.3–33.6)	<0.001
<26^+0^ weeks', *n*/*N* (%)	113/278 (40.7)	165/278 (59.4)	
26^+0^-27^+6^ weeks', *n*/*N* (%)	414/1,344 (30.8)	930/1,344 (69.2)	
28^+0^-29^+6^ weeks', *n*/*N* (%)	1,006/4,187 (24.0)	3,181/4,187 (76.0)	
30^+0^-31^+6^ weeks', *n*/*N* (%)	1,347/7,890 (17.1)	6,543/7,890 (82.9)	
32^+0^-33^+6^ weeks', *n*/*N* (%)	1,346/12,513 (10.8)	11,167/12,513 (89.2)	
Birth weight (grams), median (IQR)	1,481 (1,170–1,760)	1,657 (1,380–1,940)	<0.001
<1,000 grams, *n*/*N* (%)	514/1,432 (35.9)	918/1,432 (64.1)	
1,000–1,499 grams, *n*/*N* (%)	1,759/8,573 (20.5)	6,814/8,573 (79.5)	
1,500–2,500 grams, *n*/*N* (%)	1,887/15,670 (12.0)	13,783/15,670 (88.0)	
≥2,500 grams, *n*/*N* (%)	66/537 (12.3)	471/537 (87.7)	
Male, *n*/*N* (%)	2,638/4,226 (62.4)	12,483/21,986 (56.8)	<0.001
Small for gestational age, *n*/*N* (%)	612/4,226 (14.5)	2,796/21,986 (12.7)	0.002
1-min Apgar ≤ 3^a^, *n*/*N* (%)	416/4,024 (10.3)	762/21,184 (3.6)	<0.001
5-min Apgar ≤ 3^b^, *n*/*N* (%)	105/3,842 (2.7)	142/20,150 (0.7)	<0.001
Cesarean section, *n*/*N* (%)	2,266/4,222 (53.7)	12,185/21,976 (55.5)	0.03
Antenatal steroids^c^, *n*/*N* (%)	2,653/3,948 (67.2)	13,929/20,932 (66.5)	0.42
Prenatal care^d^, *n*/*N* (%)	4,124/4,182 (98.6)	21,504/21,789 (98.7)	0.68
Maternal hypertension^e^, *n*/*N* (%)	693/4,162 (16.7)	3,691/21,790 (16.9)	0.65
Maternal diabetes**^f^*, n*/*N* (%)	574/4,164 (13.8)	2,577/21,769 (11.8)	<0.001
Primigravida^g^, *n*/*N* (%)	1,356/4,223 (32.1)	8,029/21,966 (36.6)	<0.001
Inborn status, *n*/*N* (%)	2,691/4,226 (63.7)	15,610/21,986 (71.0)	<0.001
TRIPS score^h^, median (IQR)	19 (13–28)	12 (6–19)	<0.001
Invasive mechanical ventilation, *n*/*N* (%)	2,571/4,226 (60.8)	4,757/21,986 (21.6)	<0.001
Conventional mechanical ventilation, *n*/*N* (%)	2,349/4,226 (55.6)	4,326/21,986 (19.7)	<0.001
High frequency ventilation in the first day, *n*/*N* (%)	293/4,226 (6.9)	363/21,986 (1.7)	<0.001
EOS, *n*/*N* (%)	79/4,226 (1.9)	219/21,986 (1.0)	<0.001

*IQR, interquartile range; TRIPS, Transport Risk Index of Physiologic Stability; EOS, early-onset sepsis*.

a *Data on 1-min Apgar score were missing in 1,004 infants*.

b *Data on 5-min Apgar score were missing in 2,220 infants*.

c *Data on antenatal steroids were missing in 1,332 infants*.

d *Data on prenatal care were missing in 241 infants*.

e *Data on maternal hypertension were missing in 260 infants*.

f *Data on maternal diabetes were missing in 279 infants*.

g *Data on primigravida were missing in 23 infants*.

h *Data on TRIPS score were missing in 124 infants*.

### Trends in Cardiovascular Pharmacological Support Rate

Over the study period, there was a decreasing trend of cardiovascular pharmacological support each year. The overall cardiovascular pharmacological support rate was 18.1% (1,359/7,500), 15.9% (1,531/9,642), and 14.7% (1,336/9,070) in the first, second and third study years (χ^2^ = 35.5612, *p* < 0.001), respectively. The specific annual rate of cardiovascular pharmacological support decreased among EPI (36.3 vs. 32.4% vs. 30.5%, *p* = 0.15).

### Outcomes of Infants

The univariate analysis revealed that infants who received cardiovascular pharmacological support had higher rates of death, NEC, severe IVH, PVL, ROP, BPD, and LOS. After adjusting for relevant confounders, cardiovascular pharmacological support within the first postnatal week was independently associated with higher risks of death (aOR 3.0; 95% CI: 2.6–3.5), severe IVH (aOR 2.1; 95% CI: 1.8–2.4), and BPD (aOR 2.2; 95% CI: 2.0–2.5) (Table 2).

**Table 2:** Outcomes of infants and cardiovascular pharmacological support.

**Outcomes**	**Cardiovascular** **pharmacological support** **group, *N* = 4,226**	**No cardiovascular** **pharmacological support** **group, *N* = 21,986**	**Unadjusted odds ratio^a^** **(95% CI)**	**Adjusted odds ratio^b^ (95% CI)**
Death in first 7 days, *n*/*N* (%)	424/4,226 (10.0)	320/21,986(1.5)	7.6 (6.5–8.8)	4.2 (3.6–5.1)
Death, *n*/*N* (%)	565/4,226 (13.4)	621/21,986 (2.8)	5.3 (4.7–6.0)	3.0 (2.6–3.5)
NEC, *n*/*N* (%)	181/4,226 (4.3)	779/21,986 (3.5)	1.2 (1.0–1.4)	0.9 (0.8–1.1)
Severe IVH^c^, *n*/*N* (%)	385/3,814 (10.1)	739/20,191 (3.7)	3.0 (2.6–3.4)	2.1 (1.8–2.4)
PVL^d^, *n*/*N* (%)	134/3,656 (3.7)	479/19,041 (2.5)	1.5 (1.2–1.8)	1.0 (0.8–1.3)
ROP, *n*/*N* (%)	492/4,226 (11.6)	1,897/21,986 (8.6)	1.4 (1.3–1.6)	0.7 (0.6–0.8)
ROP ≥ stage 3, *n*/*N* (%)	68/4,226 (1.6)	149/21,986 (0.7)	2.4 (1.8–3.2)	1.1 (0.8–1.5)
BPD, *n*/*N* (%)	1,149/4,226 (27.2)	2,098/21,986 (9.5)	3.5 (3.3–3.8)	2.2 (2.0–2.5)
LOS^e^, *n*/*N* (%)	244/4,224 (5.8)	883/21,982 (4.0)	1.5 (1.3–1.7)	1.1 (0.9–1.3)

*NEC, necrotizing enterocolitis; IVH, intraventricular hemorrhage; PVL, periventricular leukomalacia; ROP, retinopathy of pre-maturity; BPD, bronchopulmonary dysplasia; LOS, culture-proven late onset sepsis*.

a *Infants in no vasopressor group served as reference*.

b *Relevant confounders adjusted for included gestational age, male, small for gestational age, 1-min Apgar score ≤3 min, cesarean section, antenatal steroids, prenatal care, maternal hypertension, maternal diabetes, primigravida, inborn status, and TRIPS score*.

c *Incidence of PVL or IVH was calculated among infants with neuroimaging results. Data on severe IVH were missing in 2,207 infants*.

d *Incidence of PVL or IVH was calculated among infants with neuroimaging results. Data on PVL were missing in 3,515 infants*.

e *Data on LOS were missing in 6 infants*.

### Treatment Duration and Outcomes

Among the 2,470 infants in whom cardiovascular pharmacological support was initiated on the first day of life, 2,450 (99.2%) had a known duration. In these patients, 5.8% (143/2,450), 16.4% (401/2,450), 14.7% (359/2,450), and 63.1% (1,547/2,450) received cardiovascular pharmacological support for 1, 2, 3 days, or more than 3 days, respectively. In total, 295 infants died during hospitalization, of whom 227 (76.9%) died while cardiovascular pharmacological treatment, leading to an abbreviated duration of therapy. Among survivors, infants who received cardiovascular pharmacological support for >3 days had lower rates of survival without morbidity (66.7 vs. 75.4%, *p* < 0.001) and higher rates of severe IVH (9.8 vs. 4.8%, *p* < 0.001) and BPD (18.7 vs. 14.1%, *p* = 0.01) compared with infants who received cardiovascular pharmacological support for ≤3 days (Table 3). After adjustment, a treatment duration >3 days was independently associated with lower rates of survival without morbidity among VLBW (*p* = 0.04; Table 4).

**Table 3:** Outcomes and cardiovascular pharmacological support duration among survivors.

**Total**	**Duration ≤ 3 days**	**Duration > 3 days**	**Odds ratio**	***p*-value**
	***N* = 722**	***N* = 1,433**	**(95% CI)**	
Survival without morbidity, *n*/*N* (%)	544/722 (75.4)	956/1,433 (66.7)	1.52 (1.24–1.88)	<0.001
NEC, *n*/*N* (%)	24/722 (3.3)	54/1,433 (3.8)	0.88 (0.51–1.46)	0.60
Severe IVH^a^, *n*/*N* (%)	32/673 (4.8)	136/1,394 (9.8)	0.46 (0.30–0.69)	<0.001
PVL^b^, *n*/*N* (%)	18/654 (2.8)	57/1,370 (4.2)	0.65 (0.36–1.14)	0.117
ROP, *n*/*N* (%)	82/722 (11.4)	204/1,433 (14.2)	0.77 (0.58–1.02)	0.06
ROP ≥ stage 3, *n*/*N* (%)	10/722 (1.4)	28/1,433 (2.0)	0.70 (0.30–1.51)	0.34
BPD, *n*/*N* (%)	102/722 (14.1)	268/1,433 (18.7)	0.72 (0.55–0.92)	0.01
LOS, *n*/*N* (%)	30/722 (4.2)	89/1,433 (6.2)	0.65 (0.41–1.01)	0.049

*NEC, necrotizing enterocolitis; IVH, intraventricular hemorrhage; PVL, periventricular leukomalacia; ROP, retinopathy of pre-maturity; BPD, bronchopulmonary dysplasia; LOS, culture-proven late onset sepsis*.

a *Incidence of PVL or IVH was calculated among infants with neuroimaging results. Data on severe IVH were missing in 49, 39 infants in 2 groups (duration ≤3 days and duration >3 days), respectively*.

b *Incidence of PVL or IVH was calculated among infants with neuroimaging results. Data on PVL were missing in 68, 63 infants in 2 groups (duration ≤3 days and duration >3 days), respectively*.

**Table 4:** Outcomes and cardiovascular pharmacological support duration in different birth weight groups among survivors.

	**Infants with birth weight ≥1,500 g**				**Infants with birth weight 1,000–1,500 g**				**Infants with birth weight < 1,000 g**			
**Outcomes**	**Duration ≤3 days**	**Duration >3 days**	**Odds ratio**	***p*-value**	**Duration ≤3 days**	**Duration >3 days**	**Odds ratio**	***p*-value**	**Duration ≤3 days**	**Duration >3 days**	**Odds ratio**	***p*-value**
	***N* = 393**	***N* = 660**	**(95% CI)**		***N* = 285**	***N* = 637**	**(95% CI)**		***N* = 44**	***N* = 136**	**(95% CI)**	
Survival without morbidity, *n*/*N* (%)	332/393 (84.5)	528/660 (80.0)	1.36 (0.96–1.93)	0.07	191/285 (67.0)	381/637 (59.8)	1.37 (1.01–1.85)	0.04	21/44 (48)	47/136 (35)	1.73 (0.82–3.64)	0.12
NEC, *n*/*N* (%)	7/393 (1.8)	10/660 (1.5)	1.18 (0.38–3.46)	0.74	15/285 (5.3)	32/637 (5.0)	1.05 (0.52–2.04)	0.88	2/44 (5)	12/136 (9)	0.49 (0.05–2.36)	0.36
Severe IVH^a^, *n*/*N* (%)	8/358 (2.2)	36/631 (5.7)	0.38 (0.15–0.84)	0.01	20/275 (7.3)	72/630 (11.4)	0.61 (0.34–1.04)	0.06	4/40 (10)	28/133 (21)	0.42 (0.10–1.32)	0.11
PVL^b^, *n*/*N* (%)	8/349 (2.3)	24/618 (3.9)	0.58 (0.22–1.35)	0.18	8/267 (3.0)	18/621 (2.9)	1.03 (0.38–2.54)	0.94	2/38 (5)	15/131 (11)	0.43 (0.05–2.00)	0.26
ROP, *n*/*N* (%)	18/393 (4.6)	29/660 (4.4)	1.04 (0.54–1.98)	0.89	48/285 (16.8)	121/637 (19.0)	0.86 (0.58–1.26)	0.44	16/44 (36)	54/136 (40)	0.87 (0.40–1.85)	0.69
ROP ≥stage 3, *n*/*N* (%)	0/393 (0)	2/660 (0.30)	0 (0–3.23)	0.28	4/285 (1.4)	12/637 (1.9)	0.74 (0.17–2.47)	0.61	6/44 (14)	14/136 (10)	1.38 (0.40–4.14)	0.54
BPD, *n*/*N* (%)	32/393 (8.1)	69/660 (10.5)	0.76 (0.47–1.20)	0.22	56/285 (19.7)	140/637 (22.0)	0.87 (0.60–1.24)	0.42	14/44 (32)	59/136 (43)	0.61 (0.27–1.31)	0.17
LOS, *n*/*N* (%)	10/393 (2.5)	16/660 (2.3)	1.12 (0.45–2.70)	0.78	19/285 (6.7)	49/637 (7.7)	0.86 (0.47–1.52)	0.58	1/44 (2)	25/136 (18)	0.10 (0.002–0.68)	0.01

*NEC, necrotizing enterocolitis; IVH, intraventricular hemorrhage; PVL, periventricular leukomalacia; ROP, retinopathy of pre-maturity; BPD, bronchopulmonary dysplasia; LOS, culture-proven late onset sepsis*.

a *Incidence of PVL or IVH was calculated among infants with neuroimaging results. Data on severe IVH were missing in 64, 18, 7 infants in 3 groups (birth weight ≥1,500 g, birth weight 1,000–1,500 g, birth weight <1,000 g), respectively*.

b *Incidence of PVL or IVH was calculated among infants with neuroimaging results. Data on PVL were missing in 87, 36, 11 infants in 3 groups (birth weight ≥1,500 g, birth weight 1,000–1,500 g, birth weight <1,000 g), respectively*.

### Discrepancy in Cardiovascular Pharmacological Support at Different NICUs

**Figure 2:**
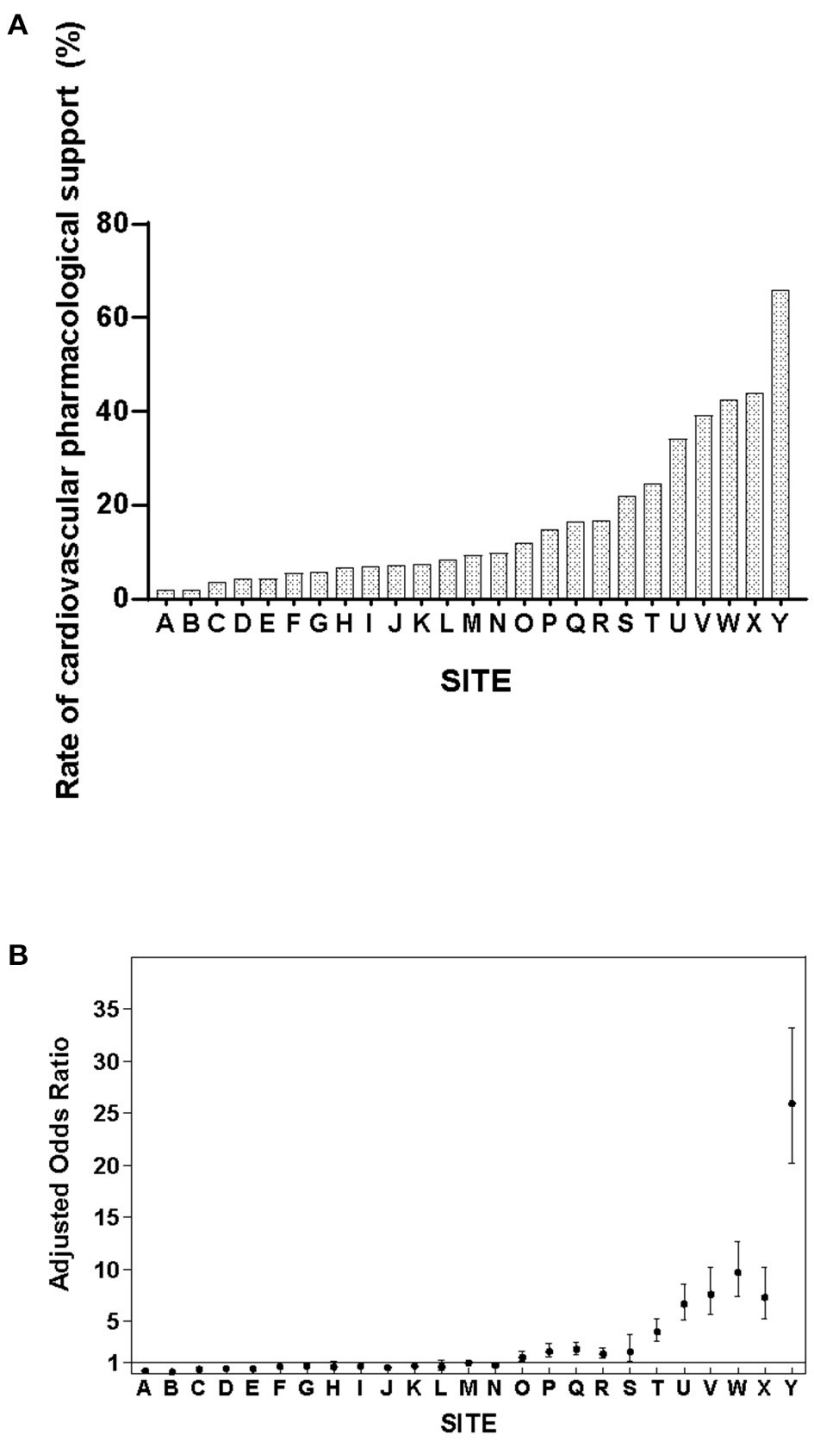
**(A)** Variability in cardiovascular pharmacological support rates among Chinese NICUs. **(B)** Adjusted odds ratios of cardiovascular pharmacological support among Chinese NICUs. Site M served as the reference site as it has the median rate of cardiovascular pharmacological support. The y-axis represents the adjusted odds ratio after controlling for confounders in multivariate regression analysis.

The rate of cardiovascular pharmacological support varied from 1.9 to 65.8% among the participating NICUs (χ^2^ = 5,100, *p* < 0.001) (Figure 2A). After adjusting for relevant confounders, the magnitude of variation remained significant (Figure 2B).

All participating centers responded to the questionnaire. The criteria to initiate cardiovascular pharmacological support varied significantly among NICUs. The most common indicators for treatment included hypotension (24/25, 96%), prolonged capillary refill time (22/25, 88%), persistent metabolic acidosis (18/25, 72%), low urine output (17/25, 68%), elevated lactate value (15/25, 60%) and diminished color of peripheries (14/25, 56%). Twenty NICUs (80%) defined hypotension as a mean blood pressure in mmHg less than the gestational age in weeks. The other definitions used were blood pressure <30 mmHg or blood pressure (in mmHg) below the 10th percentile for age and gender. All NICUs had the ability to perform invasive blood pressure monitoring and bedside cardiac ultrasound; however, targeted neonatal echocardiography (TnECHO) was not used in Chinese NICUs to assist in clinical decision making in the study period. In 22 NICUs (88%), dopamine alone was the first line drug, two hospitals used dopamine in combination with dobutamine, and one hospital used three agents together (dopamine, dobutamine, and milrinone) as the first line treatment.

## Discussion

To our knowledge, this is the largest contemporary cohort of preterm infants and the first study to evaluate cardiovascular pharmacological support in Chinese NICUs. Our results showed higher cardiovascular pharmacological support rates than those of the Norwegian Neonatal Network (NNN) and the Canadian Neonatal Network (CNN), suggesting that Chinese NICUs might overuse cardiovascular pharmacological support (19). Neonates who received cardiovascular pharmacological support, particularly those who received treatment for more than 3 days, were more likely to die or develop neonatal morbidity.

Exclusive reliance on blood pressure as the singular metric of hemodynamic stability, a lack of consensus on definition of hypotension and a lack of consensus on thresholds for therapeutic intervention led to marked variation in cardiovascular pharmacological support. Several studies have demonstrated variation in cardiovascular pharmacological support among different centers (19–21). A recent US study showed that cardiovascular pharmacological support rates ranged from 4 to 39% among six NICUs (22). Data from NNN (23) and CNN (19) also showed marked variations in cardiovascular pharmacological support rates ranging between 1–8% and 0–36% among different NICUs, respectively. Our results demonstrated the highest order of magnitude variation, specifically, a 35-fold difference (1.9–65.8%) in drug use among Chinese NICUs. A lack of reliable normative blood pressure data in preterm infants, non-judicious use of cardiovascular pharmacological support, and diagnostic imprecision related to poor access to TnECHO services may contribute to variations in practice (19–21, 24–26). Institutional habits and culture regarding cardiovascular pharmacological support may also play a role in the variation of use rate.

In recent decades, with the development of perinatal care, the number of extremely preterm infants and very preterm infants cared in Chinese NICUs has increased significantly and the experience in treatment of preterm infants have been accumulated, which may contribute to the decreasing trend of cardiovascular pharmacological support reported in our study. Previous studies have shown that antenatal corticosteroids are crucial treatment in improving neonatal outcomes and decreasing mortality for those preterm infants (27), which may be helpful to prevent the need for cardiovascular support. Although still lower than developed countries, it is still inspiriting to see an increasing trend of antenatal steroid use during the past a few years (11, 28, 29). More evidence-based practices regarding resuscitation and initial stabilization have been adopted in Chinese perinatal centers, such as delayed cord clamping and proper temperature control (30). On the other hand, from our daily clinical experience, we have observed that the concept of “treating the baby instead of treating the number (such as blood pressure)” has been increasing recognized by neonatologists in China, which may help to decrease the unnecessary use of cardiovascular pharmacological support. Further investigations are needed to interpret the reason for the decreasing trend and further optimize cardiovascular pharmacological support in Chinese NICUs.

We can also see a higher rate of cardiovascular pharmacological support in infants diagnosed with EOS which is reasonable by providing inotropic actions as well as decreasing systemic vascular resistance. Conventionally, dopamine is the first-line vasoactive drug in neonatal septic shock which is used empirical and extrapolated from adult and pediatric data mostly. It is unclear whether the treatment of low blood pressure results in any improvement of clinical outcomes or long-term neurological results. Previous studies have highlighted a potential negative relationship between cardiovascular pharmacological support and neonatal outcomes (3, 6, 31, 32). It remains unclear whether these findings relate to diagnostic imprecision or incorrect cardiovascular treatment choice, for example, vasopressor vs. inotrope. The results of our study reaffirmed early cardiovascular pharmacological support may be a marker for a clinical situation of increased risk of death and IVH, and these findings are consistent with results from the CNN (19) and the US study (9). Vasopressors may induce rapid cerebral hemodynamic changes, thereby impacting the germinal matrix, which may lead to IVH (33, 34). Other studies have proposed that vasopressors may affect the self-regulation of cerebral vessels, leading to cerebral ischemia and reperfusion injury prior to IVH occurrence (8, 35). Besides, non-judicious use of cardiovascular pharmacological support may also paradoxically augment the magnitude of left-right shunt of PDA by increasing systemic vascular resistance (36). In addition, several studies have demonstrated that although cardiovascular pharmacological support improve the numeric value of blood pressure, they may compromise systemic perfusion of vital organs and result in organ damage (9, 34, 37). These data should not dissuade neonatologists against monitoring blood pressure; rather, it is incumbent of clinicians and hemodynamic scientists to identify critical blood pressure thresholds unique to relevant pathophysiologic states and evidence for disease-specific treatment strategies. We also found an increased risk of LOS in the cardiovascular pharmacological support group which may relate to sicker infants are more likely to have end-organ complications or receive steroids which may increase the risk of LOS.

Another striking finding was the high (63.1%) rate of prolonged (>3 days) cardiovascular pharmacological support compared to a median dopamine infusion time of 46 h per patient in NNN. We identified lower rates of “survival without morbidity” in VLBW infants who received prolonged cardiovascular pharmacological support. Although a major difference was also seen in ELBW and infants with birth weight ≥1,500 infants, this was not statistically significant, which was most likely related to inadequate number of infants. The association between a high rate of cardiovascular pharmacological support in the early postnatal period and hypotension is thought to be related to impaired cardiac loading conditions due to the physiologic changes that occur in the transition environment (2, 38, 39). Dempsey et al. found a spontaneous increase in blood pressure within 24 h after birth in ELBW infants who remained untreated (40). These findings were replicated by Batton et al. (41). These findings suggest that a shorter duration of cardiovascular pharmacological support might be sufficient for transient postnatal hypotension, if needed. There is still uncertainty regarding the optimal duration of cardiovascular pharmacological support (42).

Our study did not collect specific information on the type or dose of cardiovascular pharmacological support used for individual infants; however, a *post-hoc* survey concluded that dopamine was uniformly used as the first line agent. Definitely, there is considerable controversy in the choice of cardiovascular pharmacological support and the optimal drug in different situations. Besides, there is still uncertainty according to safety and effectiveness of vasopressor activity and inotropic activity drugs as for the insufficiency of pharmacology and pharmacodynamics information in these vulnerable group. It is noteworthy that dopamine has become the universal first line agent for hemodynamic instability, irrespective of disease state or ambient cardiovascular physiology, despite few data to support this trend. Dopamine appears to elevate blood pressure predominantly by vasoconstriction at the expense of systemic blood flow. It may have negative effects on hemodynamic stability, oxygen transport, cerebrovascular autoregulation, and cardiac output through an inotrope/vasopressor imbalance (43). For cardiovascular support in preterm infants, growing data suggest that dobutamine may be more appropriate during the transitional period than dopamine (33). Whether the adverse effects on neonatal outcomes in Chinese NICUs relate to the imprecision in the prolonged use of cardiovascular pharmacological support or non-judicious use of dopamine as the first line agent remains unknown. Previous study in US showed a declining trend in the use of dopamine and dobutamine and an increasing trend of the uses of hydrocortisone and vasopressin (1). These data highlight the importance to developed standardized clinical practice guidelines. The use of TnECHO might enhance diagnostic/therapeutic precision and provide additional details for longitudinal monitoring. Further quality improvement for cardiovascular pharmacological support could be made by standardizing decision-making process, redefining intervention framework of hypotension triggering therapies, improving antenatal corticosteroids provision, shortening the duration of cardiovascular pharmacological support and develop optimal management toward transient physiological blood pressure fluctuations.

Our study has several limitations. First, this study was based on a secondary analysis of a large prospective database, and the database did not include details about the type and dosage of cardiovascular pharmacological support. In addition, we did not collect data on delayed cord clamping, PDA, or receipt of surfactant, which may play a contributory role. Second, information about indications for medication initiation and termination was also not collected. Besides, data on the occurrence time of IVH was not available leading to difficulty in clarifying the association between cardiovascular pharmacological support and IVH. Third, our study only enrolled tertiary centers located in large metropolis cities, and the results might not represent the general status of cardiovascular pharmacological support in all levels of NICUs in China. Fourth, we only collected NICU-level data in our questionnaire, but unfortunately there might be variations of practices among different providers in the same NICU.

In conclusion, our results showed that cardiovascular pharmacological support are commonly used in Chinese NICUs, especially in extremely pre-mature infants and ELBW infants. Cardiovascular pharmacological support was associated with increased risks of death, severe IVH and BPD, and prolonged use may be associated with even worse outcomes. The rate of cardiovascular pharmacological support varied significantly between participating units. Future quality improvement efforts are urgently needed to facilitate the rational cardiovascular pharmacological support in Chinese NICUs.

## Data Availability Statement

The raw data supporting the conclusions of this article will be made available by the authors, without undue reservation.

## Author Contributions

NL conceptualized, designed the study, analyzed, interpreted data, drafted the manuscript, and performed statistical analyses. SJ conceptualized, designed the study, acquired, analyzed, interpreted data, revised the manuscript, and performed statistical analyses. PM analyzed, interpreted data, reviewed, and revised the manuscript for important intellectual content. XL, YG, YW, and YY interpreted data, reviewed, and revised the manuscript for important intellectual content. JH and YD acquired, interpreted data, reviewed, and revised the manuscript for important intellectual content. SL and YC conceptualized, designed the study, analyzed, interpreted data, reviewed, and revised the manuscript for important intellectual content and supervised the study. All authors approved the final manuscript as submitted and agree to be accountable for all aspects of the work.

## Conflict of Interest

SL received support for article research from the Canadian Institutes of Health Research (CIHR). YC institution received funding from China Medical Board and CIHR. The remaining authors declare that the research was conducted in the absence of any commercial or financial relationships that could be construed as a potential conflict of interest.

## Abbreviations

NICUs: neonatal intensive care units
IVH: intraventricular hemorrhage
BPD: bronchopulmonary dysplasia
PDA: patent ductus arteriosus
TRIPS: transport risk index of physiologic stability score
PVL: periventricular leukomalacia
ROP: retinopathy of pre-maturity
EOS: early onset sepsis
LOS: late onset sepsis
NEC: necrotizing enterocolitis
EPI: extremely pre-mature infants
VLBW: very low birth weight infants
ELBW: extremely low birth weight infants
TnECHO: targeted neonatal echocardiography
NNN: Norwegian Neonatal Network
CNN: Canadian Neonatal Network.
