# Supplementary material for: Cardiovascular Pharmacological Support Among Preterm Infants in Chinese Referral Center Neonatal Intensive Care Units
Source: Front Pediatr. 2021 Apr 22;9:638540. doi: 10.3389/fped.2021.638540 (PMC8100183; doi:10.3389/fped.2021.638540)
Supplement: Supplementary file 1 [file Data_Sheet_1.DOCX]

**Appendix**

A full list of REIN-EPIQ Writing Study Group：

Siyuan Jiang, MD, Na Chen, MD, Yingying Wang, MD, and Yiding Bian, MD, Children’s Hospital of Fudan University, Shanghai, China; Xiaoyan Li, MD, Jianfang Ge, MD, Children’s Hospital of ShanXi / Women Health Center of Shanxi, Shanxi, China; Mengmeng Li, MD, Women’s Hospital of Nanjing Medical University, Jiangsu, China; Zuming Yang, MD, Suzhou Municipal Hospital, Jiangsu, China; Jinzhen Guo, MD, Liangjuan Zhang, MD, Yan Gao, MD, Northwest Women and Children’s Hospital, Shaanxi, China; Qianqian Zhou, MD, Women and Children's Hospital of Hubei Province, Hubei, China; Wenlong Xiu, MD, Xia Ouyang, MD, Baoquan Zhang, MD, Fujian Provincial Maternity and Children's Hospital/Affiliated Hospital of Fujian Medical University, Fujian, China; Hui Tang, MD, The Affiliated Shenzhen Maternity and Child Healthcare Hospital of Southern Medical University, Guangdong, China; Zhihui Rong, MD, Junping Xiao, MD, Tongji Hospital, Tongji Medical College, Huazhong University of Science and Technology, Hubei, China; Jing Yuan, MD, Qingdao Women and Children’s Hospital, Shandong, China; Dandan Pan, MD, Guiyang Maternal and Child Health Care Hospital, Guizhou, China; Jie Su, MD, Fangping Zhao, MD, Jingyun Shi, MD, Gansu Provincial Maternity and Child-care Hospital, Gansu, China; Su Lin, MD, The 2nd Affiliated Hospital and Yuying Children’s Hospital of Wenzhou Medical University, Zhejiang, China; Xuefeng Hu, MD, Haiyan Hua, MD, Shanghai First Maternity and Infant Hospital, Tongji University School of Medicine, Shanghai, China; Lichun Zeng, MD, Manmei Tu, MD, Mei Wu, MD, Jiangxi Provincial Children’s Hospital, Jiangxi, China; Yanping Zhu, MD, Xiaokang Wang, MD, Yanmei Wang, MD, First Affiliated Hospital of Xinjiang Medical University, Xinjiang, China; Qiufen Wei, MD, Yan Li, MD, Lianfang Jing, MD, The Maternal and Child Health Hospital of Guangxi Zhuang Autonomous Region, Guangxi, China; Xiaoyun Zhou, MD, Qing Han, MD, Yifan Sun, MD, Jinxin Shen, MD, Children’s Hospital of Nanjing Medical University, Jiangsu, China; Shuhua Liu, MD, Children’s Hospital of Hebei Province, Hebei, China; Renqiang Yu, MD, The Affiliated Wuxi Maternity and Child Health Hospital of Nanjing Medical University, Jiangsu, China; Xianghong Liu, MD, Qilu Children’s Hospital of Shandong University, Shandong, China; Huiqing Sun, MD, Zengyuan Yu, MD, Children’s Hospital Affiliated to Zhengzhou University, Henan, China; Jinwen Weng, MD, Beijing Children’s Hospital of Capital Medical University, Beijing, China; Yanjuan Tan, The Third Xiangya Hospital of Central South University, Hunan, China.
