# Supplementary material for: Cardiovascular Pharmacological Support Among Preterm Infants in Chinese Referral Center Neonatal Intensive Care Units
Source: Front Pediatr. 2021 Apr 22;9:638540. doi: 10.3389/fped.2021.638540 (PMC8100183; doi:10.3389/fped.2021.638540)
Supplement: Supplementary file 2 [file Table_1.DOCX]

2155 infants with a clear cardiovascular pharmacological support duration

27532 infants with gestational age <34 weeks

1320 infants excluded

1320 infants discharged against medical advice

26212 eligible infants

4226 infants with cardiovascular pharmacological support

21986 infants without cardiovascular pharmacological support

2470 infants with cardiovascular pharmacological support initiated on the first day

20 infants with an unknown duration

295 fatal cases with an unclear duration

Subgroup analysis

Duration ≤3 days: 722 infants

Duration >3 days: 1433 infants

Duration ≤3 days: 44 infants

Duration >3 days: 136 infants

Infants with birth weight ≥1500 g

Duration ≤3 days: 393 infants

Duration >3 days: 660 infants

Infants with birth weight 1000-1500 g

Duration ≤3 days: 285 infants

Duration >3 days: 637 infants

Infants with birth weight <1000 g
