# Supplementary material for: Cardiovascular Pharmacological Support Among Preterm Infants in Chinese Referral Center Neonatal Intensive Care Units
Source: Front Pediatr. 2021 Apr 22;9:638540. doi: 10.3389/fped.2021.638540 (PMC8100183; doi:10.3389/fped.2021.638540)
Supplement: Supplementary file 3 [file Table_2.DOCX]

Supplement

Investigation of cardiovascular pharmacological support in NICUs

1. Which is the most commonly used cardiovascular cardiotrope in the first postnatal week in infants<34 weeks gestation in your unit? If you commonly use the combination of 2 or more than 2 cardiotropes together in your site, what is your therapeutic option? (single choice)

Dopamine  Dobutamine  Epinephrine  Norepinephrine  Milrinone

Combination of 2 or more than 2drugs (please list your therapeutic option)

1. What are the indications for cardiovascular pharmacological support in the first postnatal week in infants<34 weeks gestation according to your unit? (multiple choice)

Hypotension ☐ Low urine output  Prolonged capillary refill time

Diminished color of peripheries  Tachycardia  Elevated lactate value

Persistent metabolic acidosis Hemodynamic instability according to echocardiography

☐ Other indications (please list the indication)

1. Is invasive blood pressure monitoring available in your hospital? (single choice)

☐ Yes ☐ No

4. Which is commonly used diagnostic criteria of hypotension according to preterm infants in your site? (single choice)

Hypotension is defined as a mean blood pressure in mmHg less than the gestational age in weeks.

Hypotension is defined as blood pressure less than 30 mmHg.

Hypotension is defined as blood pressure (in mmHg) below the 10th percentile for age and gender.

Other definitions (please list the definition)

5. What are the indications for discontinuing cardiovascular pharmacological support in the first postnatal week in infants<34 weeks gestation according to your unit? (multiple choices)

Normal blood pressure  Normal urine output  Normal capillary refill time

Ruddy of peripheries  Normal heart rate  Normal lactate value

Correction of metabolic acidosis  Hemodynamic stability according to echocardiography

Other indications (please list the indication)

6. Is bedside cardiac ultrasound available in your unit?

Yes  No

7. Is targeted neonatal echocardiography (TnECHO) available in your unit? If the answer is yes, do you use TnECHO to enhance diagnostic / therapeutic precision?

Yes  No
